# Editorial: Non Genomic Actions of Thyroid Hormones in Cancer

**DOI:** 10.3389/fendo.2019.00847

**Published:** 2019-12-04

**Authors:** Paul J. Davis, Osnat Ashur-Fabian, Sandra Incerpi, Shaker A. Mousa

**Affiliations:** ^1^Pharmaceutical Research Institute, Albany College of Pharmacy and Health Sciences, Rensselaer, NY, United States; ^2^Department of Human Molecular Genetics and Biochemistry, Sackler School of Medicine, Tel Aviv University, Tel Aviv, Israel; ^3^Department of Sciences, University Roma Tre, Rome, Italy

**Keywords:** integrin αvβ3, angiogenesis, anti-apoptosis, thyroxine, signal transduction, driver genes, tumor microenvironment, tetrac

The molecular basis of the actions of thyroid hormone requires cellular uptake and liganding of the hormones by specific receptors in the cell nucleus and consequent expression of certain genes (1). That another panel of thyroid hormone receptors might allow iodothyronines to act without entering the cell appeared to be unlikely until a structural protein of the plasma membrane of rapidly dividing endothelial cells and of cancer cells (1, 2) was found to have a discrete receptor for thyroid hormone analogs. This receptor is on the extracellular domain of integrin αvβ3. It enables thyroid hormone stimulation of tumor cell proliferation, of tumor-linked angiogenesis, of tumor cell defense mechanisms, e.g., anti-apoptosis, and, apparently, of chemoresistance and radioresistance (Cayrol et al.; Davis et al.; Krashin et al.). A series of eight publications in a recent issue of *Frontiers in Endocrinology* is devoted to “Integrin αvβ3, non-peptide hormones and cancer” (Ashur-Fabian et al.; Cayrol et al.; Chin et al.; Davis et al.; Gionfra et al.; Hercbergs; Krashin et al.; Uzair et al.).

This previously unrecognized set of mechanisms of actions of thyroid hormones—initiated at the cell surface—has clinical implications (Hercbergs). The principal ligand for the hormone receptor on αvβ3 is L-thyroxine (T4) [(2), Davis et al.]. 3,5,3′-triiodo-L-thyronine (T3) (Uzair et al.) and reverse T3 (rT3) (Davis et al.) may have limited actions at the integrin, but Hercbergs has shown that pharmacological elimination of host T4 and substitution of T3 can serve to arrest tumor growth.

Transcription of an extensive panel of genes may be differentially regulated by T4 at its receptor on αvβ3 of cancer cells (Cayrol et al.; Davis et al.); these include driver genes and genes involved in signal transduction (2), as well as the processes of angiogenesis (Cayrol et al.) and apoptosis [(2), Davis et al.] mentioned above, and epithelial-mesenchymal transition (EMT) (Uzair et al.) and the state of cellular actin (Uzair et al.).

The *Frontiers in Endocrinology* papers on αvβ3 and thyroid hormone also broaden the spectrum of cancers subject to control from this site. The growth of melanoma, particularly that of the eye, may be arrested via tetraiodothyroacetic acid (tetrac), a deaminated T4 analog that inhibits T4 actions at the integrin (Ashur-Fabian et al.). Proliferation of T cell lymphoma cells and angiogenesis at sites of lymphoma xenografts are subject to regulation via αvβ3 (Cayrol et al.). Interestingly, *K-RAS* status (wild vs. mutant) in human colorectal cancer (CRC) cells alters the abundance of heterodimeric αvβ3 protein, but the relatively low threshold for tetrac/integrin activity at the integrin is unchanged by *K-RAS* status and tetrac and chemically modified tetrac are equally effective in mutant and wild-type CRC (Chin et al.).

It is important to note that normal, non-malignant cells express reduced quantities of αvβ3 and the conformation of the integrin—a reflection of its state of activation (2)—is such that its signaling functions appear to be limited. Integrin αvβ3 is one of the more than two dozen heterodimeric integrins that are structural proteins of the plasma membrane, serving a variety of functions by interacting with proteins and other cells in the immediate microenvironment. The microenvironment specific for αvβ3 is extracellular matrix proteins (vitronectin, fibronectin, osteopontin, etc., containing the Arg-Gly-Asp [RGD] sequence) and various growth factor receptors (1). In a single cell, αvβ3 may also interact with adjacent vascular growth factor receptors. All such interactions in the immediate cellular environment may cause αvβ3 and other integrins to activate intracellular signal transduction pathways with specific downstream consequences in terms of gene expression and cell function. We have not identified other integrins to contain thyroid hormone receptor sites (HY Lin: unpublished observations).

The significance of the recognition of the existence of the receptor for thyroid hormone and perhaps for other non-peptide hormones includes: (1) the receptors are new therapeutic targets for which ligands already exist, e.g., tetrac or chemically modified tetrac as an antagonist of T4; (2) scanning of tumors is feasible with radiolabeled T4, modified structurally so that it does not enter the cells, and (3) drug delivery to tumors with tetrac coupled to a drug-binding nanoparticle, such as poly-lactic-co-glycolic acid, is possible.

In conclusion, it is clear that thyroid hormone as T4 can support cancer growth via cell surface αvβ3. Exploitation of these receptors may have useful therapeutic consequences.

## Author Contributions

PD drafted the editorial. All authors contributed to manuscript revision, read, and approved the submitted version.

### Conflict of Interest

The authors declare that the research was conducted in the absence of any commercial or financial relationships that could be construed as a potential conflict of interest.
